# Weight Bias Internalization as an Embodied Process: Understanding How Obesity Stigma Gets Under the Skin

**DOI:** 10.3389/fpsyg.2019.00953

**Published:** 2019-04-29

**Authors:** Oli Williams, Ellen Annandale

**Affiliations:** ^1^University of Leicester, Leicester, United Kingdom; ^2^Collaboration for Leadership in Applied Health Research and Care East Midlands, University of Nottingham, Nottingham, United Kingdom; ^3^University of York, York, United Kingdom

**Keywords:** obesity, embodiment, stigma, internalization, health

## Introduction

This *Opinion Article* contributes to this Special Issue a supportive critique of the weight bias internalization analysis. The explicit aim is to broaden the ways in which “internalization” is currently defined and analyzed in research on weight bias and to encourage interdisciplinary research endeavors to increase our understanding of its implications. Both authors are sociologists who understand and analyse the individual condition as embodied^1^. In short, we are interested in how and in what ways the social world “gets under the skin” and thus has psychosomatic implications. It is for this reason why, despite commending much of the scholarship on weight bias internalization and accepting the validity of the research findings, we feel it necessary to challenge the current application of the “internalization” terminology. Our argument is that weight bias internalization research is limited in that it is largely disembodied. This is considered problematic because to fully understand the *implications* of weight bias internalization (the express concern of this Special Issue), it is necessary to appreciate both *how* and *in what ways* it gets under the skin.

## Weight Bias: Discriminatory, Detrimental and Counter-Productive

Weight bias and obesity stigma are terms commonly used synonymously in research literature. Both describe a cultural framing that emphasizes the role of individual behavior or “lifestyle” as the cause and cure of “obesity” (Crossley, 2004) and forefronts an economic rationale to moralize and individualize the issue (i.e., implying that those who are classified as “overweight” and/or “obese”^2^ are irresponsible individuals who are placing an avoidable burden on national health systems). As evidenced elsewhere (Williams and Annandale, 2018), our analysis sits within the tradition of Critical Obesity Studies recognizing that (i) obesity and overweight are socially constructed clinical categories—established via the Body Mass Index (BMI)—which offer crude and flawed indicators of health and (ii) the metabolically healthy but obese phenotype has been demonstrated empirically with physical activity and diet (independent of weight) acting as more reliable indicators of overall health status than BMI (e.g., Ortega et al., 2013). We endorse Rich's (2011, p. 16) argument that dominant cultural framings of people of higher weights “not only position individuals as blameworthy, but moralize and decontextualize health inequalities by glossing over the social and structural contexts.” Because obesity has been consistently shown to follow social gradients in wealth and inequality (e.g., Pickett et al., 2005; Krueger and Reither, 2015; Baker, 2018), this depiction is often tantamount to victim blaming. Even outside of the influence of social inequalities, the logic of motivating people to comply with official health guidelines by moralizing behaviors and promoting the internationalization of weight-based stigma is highly questionable (see e.g., LeBesco, 2011; Täuber et al., 2018). Therefore, the implications of weight bias/stigma are important from both health promotion and social justice perspectives and have thus predictably become significant research inquiries.

The study of weight bias/stigma is a truly multidisciplinary field and despite significant disciplinary differences, the research findings are characterized by coherence. Review articles have concluded that not only is weight bias/stigma an ineffective means by which to reduce the incidence of obesity but that it actually promotes weight gain and has additional iatrogenic consequences (Puhl and Heuer, 2009; Brewis, 2014; Rees et al., 2014; Phelan et al., 2015). Weight bias/stigma has been shown to have significant detrimental mental health and behavioral implications, e.g., increasing vulnerability to stress, depression, low self-esteem, poor body image, maladaptive eating behaviors, and exercise avoidance (Hayward et al., 2018; Tomiyama et al., 2018; Tomiyama, 2019). Tomiyama et al., 2018 review outlines the rapidly growing evidence base that indicates the detrimental impact of weight bias/stigma cannot simply be explained away by higher bodyweights leading to poorer health and/or greater likelihood of perceiving weight-related discrimination. Rather, the review highlights that negative characterization of people classified as overweight/obese has led to simply *perceiving* oneself as overweight to have a prospective association with biological markers of poorer health. Research is in its infancy but findings indicate that the biological implications of weight bias/stigma range from increased secretion of the fat-storage promoting stress hormone cortisol (Jackson and Steptoe, 2018) to higher risk of developing dementia (Sutin et al., 2018); the former linked to the common practice of yo-yo dieting/weight cycling and its associated adverse health effects (Tomiyama, 2014; Madigan et al., 2018). Additionally, weight bias/stigma has been shown to translate into structural inequities, e.g., in employment, healthcare, and education, which reproduces the social disadvantage that drives the uneven distribution of obesity incidence throughout the socioeconomic spectrum (Puhl and Heuer, 2009; Tomiyama, 2019).

Puhl and Heuer (2010) argue that to improve public health it is essential that common societal assumptions that perpetuate weight bias/stigma are challenged and that the deleterious repercussions of weight bias/stigma inform the ways in which obesity is popularly framed and understood. Some will be unconcerned with the ethics of stigma and the imperative to reduce human suffering. However, they may be convinced of the need for change by the evidence demonstrating the ineffective, counter-productive and detrimental outcomes of weight bias/stigma. Consequently, it is vital that researchers analyse the processes through which the sociocultural phenomenon of weight bias/stigma affects people's health; that is, how the social (weight bias/stigma) gets under the skin. Or put another way, the processes by which external social factors are internalized and the psychosomatic consequences that follow. Despite the necessity of this inquiry, when moving from research on weight bias/stigma more generally to the more specific inquiry of weight bias internalization the field is limited in what it can offer. Therefore, it is necessary to critically evaluate the theoretical and methodological traditions that define the study of weight bias internalization.

## Getting Under the Skin: Internalization as Embodiment

Weight bias internalization has been defined as the “internalization of negative weight stereotypes and subsequent self-disparagement” (Pearl and Puhl, 2018, p. 1141). In short, over time people who are classified as overweight or obese come to accept and endorse derogatory and discriminatory cultural depictions of people who are classified as overweight or obese (i.e., as irresponsible, gluttonous, and lazy). A systematic review of the literature has demonstrated that the empirical study of this phenomenon is in its infancy (Pearl and Puhl, 2018). But early findings illustrate a significant detrimental impact on mental (e.g., depression, anxiety, body dissatisfaction) and physical health (e.g., metabolic syndrome, weight cycling) as well as related health behaviors through rejection of dietary advice, binge eating and exercise avoidance (Ratcliffe and Ellison, 2015; Jackson and Steptoe, 2017; Puhl and Himmelstein, 2018). These are useful and important findings, but in such studies internalization is defined and analyzed predominantly as a cognitive process. Indeed, the *Weight Bias Internalization Scale* is “a measure of belief in social stereotypes relating to obesity and negative self-evaluations due to one's weight” (Durso and Latner, 2008, p. 81). This is an unnecessarily restrictive definition of “internalization” that unhelpfully narrows the parameters of inquiry.

While finding that self-blame and behaviors considered detrimental to one's health result from both self-endorsement of anti-fat attitudes and accepting weight-based stereotypes is certainly one element of weight bias internalization (i.e., a psychological component which subsequently influences patterns of behavior), it is only that: one component of a far more complex process of internalization. However, this component has come to define the field. This helps explain why the only systematic review of this literature (Pearl and Puhl, 2018) found that while there is evidence of strong, negative relationships between weight bias internalization and mental health outcomes, few studies have examined the relationship between weight bias internalization and physical health. Succinctly put, the predominance of psychological analysis is skewing the field.

On the basis of existing research it is fair to argue that in the study of weight bias internalization cognition has taken precedence over other interrelated processes. Given that weight bias has been shown to have social and biological implications, the delimited focus in the field on a cognitive process seems unjustified. The predominantly cognitive component that is currently most commonly referred to and researched as weight bias internalization could perhaps more accurately be referred to as weight bias “endorsement,” “acceptance,” or “agreement.” This would better reflect its partial (albeit important) role in the broader biopsychosocial process of weight bias internalization—which could alternatively be defined as: detrimental psychosomatic responses caused by exposure to discrimination on the basis of negative stereotypes about people of higher weights. An interdisciplinary approach to understanding weight bias internalization as an embodied phenomenon would go some way to painting a more complete picture of the implications of weight bias internalization.

Crossley (2006, p. 2) succinctly summarizes “reflexive embodiment” by explaining that “human bodies exist in two dimensions. We are our bodies (being) but sometimes perceive them as an object that we possess (having).” Bodies are both subjectively (personally) and objectively (materially) experienced. This is how weight bias/stigma (an external/social factor) can make bodies *feel* particular ways through psychosomatic experience and initiate changes in biological markers of health. For Freund (2011), this is because mind and body do not operate separately but rather we are all “mindbodies” with the potential to self-initiate health states on a conscious-unconscious level. It is this interconnected and dependent relationship between mental and physical, cognitive and carnal, which accounts for the well-established “placebo effect,” but it also explains how the social gets under the skin. For instance, on top of the physical repercussions of related behaviors (e.g., maladaptive eating behaviors and exercise avoidance), this is how the depression someone may experience as a result of being/having a stigmatized body can detrimentally impact their physical health (through, for instance, the secretion of cortisol and associated effects—see e.g., Tomiyama, 2019). An embodied analysis of weight bias internalization needs to be inclusive of, but to extend beyond cognitive processes and their behavioral consequences by appreciating the biopsychosocial mechanisms through which weight bias is internalized and has consequence. That is, how the social (weight bias/stigma) comes to have material implications (detrimental health impacts).

Social scientists, particularly those employing a feminist analysis, have been at the forefront of establishing an embodied analysis of obesity (e.g., Bordo, 1993; Murray, 2012; Warin, 2015; Lupton, 2018). This work demonstrates the limits of approaching the study of obesity as primarily a biological, psychological or social and political phenomenon instead highlighting the inextricable interplay of these constituent factors and strengthens the analysis, methodology, and ethics of weight bias/stigma research. For example, the research trend of putting thin people in “fat suits” has been critiqued by Meadows et al. (2017) who highlight the impossibility of replicating the physiological, affective, and behavioral responses to weight stigma outside of the embodied experiences of people of higher weights. Furthermore, it is increasingly appreciated that the physical body does not simply respond to external social forces, but dynamically engages with them in an iterative process (see e.g., Barad, 2007). For example, in her book *Gut Feminism* Wilson (2015) explores the “biological enactments” of bulimia—a condition not uncommon amongst people categorized as overweight and obese (Brownwell and Walsh, 2017)—to think anew about the mind-body relationship. As she explains, especially where girls and women are concerned, bulimia is commonly accounted for as an ideational response to living within patriarchal societies; a visceral response as the individual “wills” the food back up by induced vomiting (antiperistalsis). Wilson (2015, p. 62) argues that in chronic cases, “organic thought” occurs as binging and vomiting become compulsive and not necessarily tied to consciously meaningful and analyzable events in a person's social world. Thus, she argues that the “organism itself is beginning to think” as distress and anger become “primarily organic.”

We have used an embodied analysis in a study of three weight-loss groups in England to highlight how obesity stigma can confuse people's objective and subjective experiences of their bodies (Williams and Annandale, 2018). Confusion was primarily evident on occasions when group members *felt* heavier after engaging in negatively moralized behaviors associated with weight-gain but this “weight” did not register on the weighing scales. We conceptualize this as the *weight of expectation* which we take as illustrative of how the morality that characterizes weight-management within a culture that is hostile to those categorized as overweight or obese gets under the skin and is *felt* in the flesh. An embodied analysis allowed us to pay attention to and take seriously an implication of weight bias that would otherwise be unobservable via the *Weight Bias Internalization Scale*. Analyzing the embodiment of obesity stigma also allowed us to demonstrate how weight-loss group participants came to ascribe their experiences of sensations deriving from physiological responses to exercise (e.g., sweating, delayed onset muscle soreness) with positive moral and social significance. These *carnal cues* played an important role in their attempts to negotiate obesity stigma and illustrate how the effects of weight bias extend beyond verbal or written endorsement of discriminatory anti-fat attitudes to include interpretations of physiological processes and bodily sensation. These findings are important to the analysis of the implications of weight bias internalization because they deepen understanding of the lived experience of being stigmatized as well as how and why obesity stigma is an inappropriate and ineffective means of promoting weight-loss and health. However, they are not and cannot be researched within the parameters, and via the methods, established for the study of weight bias internalization. The same is true of the biological markers of poorer health attributable to weight bias internalization.

We contend that an embodied analysis would go some way to necessarily extending the analysis of weight bias internalization but, of course, we do not claim to have all the answers, as no one discipline can. Instead, we argue for an opening up of the definition and analysis of weight bias internalization so as to fully appreciate and measure the implications of weight bias/stigma. This is necessarily an interdisciplinary endeavor. In a demonstration of the potential utility of interdisciplinarity in this field, Tomiyama (2014) took a biopsychosocial approach to create a generative model to explain how and why weight-based stigma is counter-productive: the cyclic obesity/weight-based stigma (COBWEBS) model. The model depicts a “vicious cycle”—with people getting “caught” in COBWEBS—wherein weight-based stigma is characterized as a stressor that begets weight gain through increased eating and other biobehavioral mechanisms (e.g., elevated secretion of cortisol and associated fat storage). This appears to us to be a useful model through which to engage with revealing one of the biopsychosocial implications of weight bias internalization, but to test its fidelity requires the kind of interdisciplinary research that is as yet outside the scope of current definitions and measures used in this field of study.

## Conclusion

The dominant definition of weight bias internalization and the associated methods for measuring its effects are limited and thus risk rendering its full impact immeasurable to those working in the field. Relatedly, they elevate mind and rational decision making over body and psychosomatic sensation. Ironically this has the effect that current analysis reveals very little about the process of internalization understood more broadly as a biopsychosocial process—that is, how weight bias quite literally gets under the skin. Presently a preoccupation with internalization as a psychological process has generated evidence that tells us far more about changes in states of mental health and behavioral outcomes than about biological effects and the lived experience of obesity stigma as mediated through the body. This is not to denigrate this contribution, on the contrary, it has greatly advanced understanding of the implications of weight bias. However, developing and strengthening the evidence base now relies upon broadening the definition of internalization to foster the interdisciplinarity necessary to realize the biopsychosocial analysis required to fully comprehend the implications of weight bias.

## Author Contributions

OW and EA both contributed more or less evenly to the formation of the theoretical argument and the writing of the manuscript.

### Conflict of Interest Statement

The authors declare that the research was conducted in the absence of any commercial or financial relationships that could be construed as a potential conflict of interest.

## Footnotes

^1^We have defined embodiment elsewhere as depicting “the fusion of the mind and body in a process whereby the society and culture within which we live are experienced in bodily terms and internalized by us: they are embodied” (Williams and Annandale, 2014, p. 1868).

^2^We initially place these terms in inverted commas in recognition that their meaning and use are challenged. In this article these terms are not referred to uncritically but are used to be consistent with the wider literature.
